# Neural Correlates of Outcome Anticipation in Multiple Sclerosis

**DOI:** 10.3389/fneur.2018.00572

**Published:** 2018-07-25

**Authors:** Angela Spirou, Pei-Pei Liu, Joman Y. Natsheh, Eliane Neuteboom, Ekaterina Dobryakova

**Affiliations:** ^1^Traumatic Brain Injury Research, Kessler Foundation, East Hanover, NJ, United States; ^2^Department of Physical Medicine and Rehabilitation, Rutgers New Jersey Medical School, Newark, NJ, United States; ^3^Neuropsychology and Neuroscience Research, Kessler Foundation, East Hanover, NJ, United States; ^4^Department of Anatomy & Neurosciences, University of Amsterdam, Amsterdam, Netherlands

**Keywords:** fMRI, reward, anticipation, multiple sclerosis, striatum, hippocampus, motivation

## Abstract

Outcome anticipation is not only a mental preparation for upcoming consequences, but also an essential component of learning and decision-making. Thus, anticipation of consequences is a key process in everyday functioning. The striatum and the ventromedial prefrontal cortex are among the key regions that have been shown to be involved in outcome anticipation. However, while structural abnormalities of these regions as well as altered decision-making have been noted in individuals with multiple sclerosis (MS), neural correlates of outcome anticipation have not been explored in this population. Thus, we examined the neural correlates of outcome anticipation in MS by analyzing brain activation in individuals with MS while they performed a modified version of a card-guessing task. Seventeen MS and 13 healthy controls performed the task while functional magnetic resonance imaging (fMRI) was obtained. To achieve maximal anticipatory response and prevent the possibility of differential performance on the task, participants were presented with monetary rewards only on 50% of the trials. While replicating previous evidence of structural abnormalities of the striatum in MS, our results further showed that individuals with MS exhibited greater activation in the putamen, right hippocampus, and posterior cingulate cortex during outcome anticipation compared to healthy controls. Furthermore, even though there was no strategy that participants could learn in order to predict outcomes, 76% of participants with MS indicated that they used strategies while performing the task. We thus propose that the increased neural activation observed in MS during outcome anticipation might be explained by a failure in recognizing the lack of regularity in the task structure that could result in using strategies to perform the task.

## Introduction

Multiple sclerosis (MS) is a neuro-inflammatory disease characterized by the demyelination and neurodegeneration of the central nervous system (1, 2). Such neuronal damage can result in symptoms commonly seen in MS, such as difficulties in vision and ambulation, numbness, dizziness, and changes in affect and cognition. Indeed, a wide range of cognitive changes can occur following MS, including changes in attention, memory, and sensory information processing. Specifically, the process of learning, which gives the ability to acquire new information and modulate behavior accordingly, has been reported to be impaired in MS (3, 4).

Learning entails building action-outcome associations. The initial anticipation of what will happen in the future is an important component of action-outcome associations, since it indicates that an organism is aware of the link between an action and an outcome, and is prepared to process the outcome caused by the action. Thus, impairments in such learning can lead to altered outcome anticipation, which in turn can interfere with adaptive decision-making 5, 6. Altered decision making has been previously reported in individuals with MS (7–10). Specifically, several studies found that individuals with MS not only made poorer decisions while trying to maximize rewards, but were also less sensitive to the risks associated with their decisions. Such poor decision-making suggests the possibility that outcome anticipation in MS might be impaired. However, the process of outcome anticipation in MS has yet to be fully examined.

The striatum, the primary nucleus of the basal ganglia (BG) which can be subdivided into the caudate nucleus and putamen, has been linked to learning, decision-making, and outcome anticipation (11–13). The striatum is topographically connected with the prefrontal cortex (PFC), with specific regions of the PFC being engaged in outcome anticipation and decision-making, such as the ventromedial prefrontal cortex (vmPFC). While it has been shown that individuals with MS exhibit structural abnormalities in these regions (14, 15), the neural correlates of outcome anticipation in MS have not been examined.

Thus, the current study examined the neural correlates involved in outcome anticipation in individuals with MS. We expected that individuals with MS will show altered activation during outcome anticipation compared to healthy controls. We also assessed motivational tendencies in our participants because of the close relationship between outcome anticipation and motivation. In addition, we controlled for depressive symptomatology, as depression frequently occurs in MS (16), and has been shown to interfere with outcome anticipation processes (17, 18).

## Methods

### Participants

Magnetic resonance imaging (MRI) and functional MRI (fMRI) data of 17 individuals with MS and 13 healthy controls (HCs) from a previous study (19) were used for analysis (Age_MeanMS_ = 44.81 [*SD* = 8.82] Age_MeanHC_ = 37.85 [*SD* = 12.58]). MS participants had no other neurological or psychiatric disorders besides MS; HC participants had no neurological or psychiatric disorders. All participants were female in order to more accurately study the population most frequently affected with MS. Participants had no history of substance or drug abuse, no MRI contraindications, and were all right handed. To avoid the effect of aging, only participants under the age of 65 were recruited. In the MS group, 16 participants were diagnosed with relapsing-remitting, and one with secondary progressive MS. The mean disease duration for the MS group was 14.53 years (*SD* = 7.76). Participants with MS were at least 4 weeks post most recent exacerbation and use of steroids, benzodiazepines, or neuroleptics. Furthermore, due to the high prevalence of depression in MS populations, the MS and HC groups were screened for depressive symptomatology by the Chicago Multiscale Depression Inventory (CMDI). The two groups showed no differences in CMDI scores. Demographic information and CMDI scores are shown in Table 1.

**Table 1:** Demographic information and depressive symptomology of both groups.

	**Groups**		
**Variable**	**HC**	**MS**	**Sig**
Gender	13 Female	17 Female	–
Age	37.85 (12.58)	44.81 (8.82)	*p* = 0.09
Education	16.15 (2.41)	15.69 (2.55)	*p* = 0.43
MS duration (years)	–	14.53 (7.76)	–
MS type			
Relapsing remitting	–	16	–
Secondary progressive	–	1	–
CMDI	49.15 (11.92)	48.88 (19.70)	*p* = 0.97

*Data shown as Mean (Standard Deviation); CMDI, Chicago Multiscale Depression Inventory*.

The study was approved by the Institutional Review Board of Kessler Foundation and is in accordance with the ethical standards laid down in the 1964 Declaration of Helsinki and its later amendments. All participants signed an informed consent and received a compensation of $50 as well as a $5 bonus from the card-guessing task.

### Behavioral paradigm

**Figure 1:**
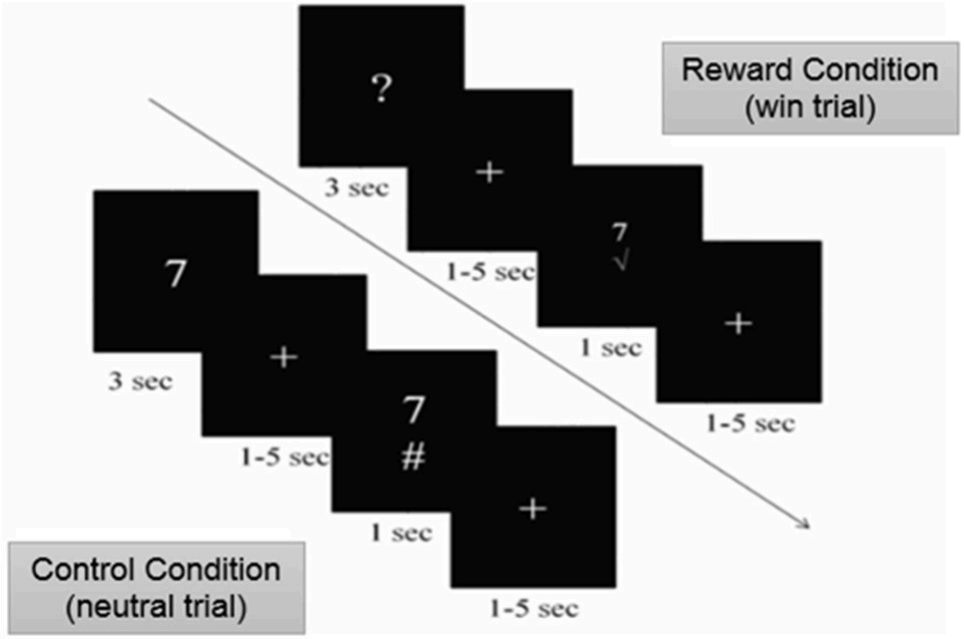
Card-Guessing Task paradigm shown to participants within MRI. During the reward condition, participants were asked to guess if a card's value was more or < 5 when prompted with a question mark. If guessed correctly, participants received a green check mark, indicating the win of $1.00 (shown above). If guessed incorrectly, participants received a red X mark, indicating a loss of $0.50. During the control condition, participants were shown numbers and indicated whether or not the number was higher or lower than five (shown above).

We examined brain activity in individuals with MS while they performed a modified version of the card-guessing task (20) with two types of conditions: the reward and control (no reward) conditions. During the reward condition, participants were told that they can win a monetary bonus if they correctly guess a number on a card. Participants were presented with monetary rewards only on 50% of the trials. This was done in order to achieve maximal unpredictability of rewarding outcomes necessary to motivate gambling and elicit anticipatory response in the brain (21, 22) as well as to eliminate the confound of differential task performance on brain activity. Specifically, during the reward condition of the task, a card with a question mark was presented on the screen (Figure 1). Participants were told that the number on the card could range from one to nine (but would never be five), and to make a guess of whether the number on each card was higher or lower than five. Participants made their guess by pressing either the left or right button on a response box placed under their right hand within 3 s. After participants responded, a blank screen with a fixation point was presented for a randomly determined period of time between 1 and 5 s (i.e., anticipation phase). After the anticipation phase, participants were presented with a green checkmark for correct guesses and a red “X” for incorrect guesses, both accompanied with the presentation of the correct number. Participants earned $1.00 reward for each correct guess and lost $0.50 for each incorrect guess. Unknown to the participants, the outcome on each card was predetermined after they made the guess, so that participants' guesses were correct on half of the trials and incorrect on the other half of the trials. Therefore, participants could not learn to predict the value of the cards and would simply be waiting to find out about the result during the anticipation phase. The procedure of this task was the same in the control condition with one exception. Instead of guessing the values on the cards, participants were presented with the values of the cards. They then used the response box to indicate whether the value on the card was higher or lower than five, a condition in which the participant only confirmed the card's value. There was no monetary rewards or losses after each trial in the control condition. The order of the runs and trials within each run was randomized across participants using E-Prime software (23).

Every participant performed two runs of the reward condition and two runs of the control condition. Each run consisted of 60 trials. Every participant received a fixed bonus of $5 at the end of the study.

After the scan, participants filled out questionnaires to measure motivational tendencies and depression. First, we used the Behavioral Inhibition System and Behavioral Activation System scales (BIS/BAS) (24, 25), which consists of 24 statements, measuring both appetitive motivation (i.e. reward responsiveness, fun seeking and drive; BAS scale) and aversive motivation (BIS scale). The Chicago Multiscale Depression Inventory (26) was then administered to gain an overall score of depressive symptomatology in order to control for differences in depression between groups.

### Data acquisition

All MRI data was acquired on a Siemens Skyra 3.0T scanner equipped with a standard 20-channel radio-frequency head coil. Thirty-two slices were obtained in an oblique orientation of 30° to the anterior commissure-posterior commissure line to prevent signal drop-out from the prefrontal cortex (27).

High-resolution structural images were collected using a standard T1-weighted pulse sequence (*TR* = 2,100 ms, *TE* = 3.43 ms; 32 slices; 1 × 1 × 1 mm voxels). Using these images, functional activation could be localized. Functional data for four runs, consisting of 172 volumes each, was collected by using a standard T2^*^-weighted echo planar sequence (*TR* = 2,000 ms; *TE* = 30 ms; 3 × 3 × 3 mm voxels; flip angle = 90°; FoV = 256 mm; slice gap = 1 mm) sensitive to the blood oxygen level dependent (BOLD) contrast.

### Data analysis

#### fMRI data

FMRIB Software Library (FSL; www.fmrib.ox.ac.uk/fsl) v5.0 was used to analyze data (28). The FMRI Expert Analysis Tool (FEAT) v6.00 was used for general linear model-based fMRI analysis. Data passed through a high pass filter of 90 s to keep out low frequency noise. The brain extraction tool was used to skull strip raw BOLD images (29). The effect of head motion was controlled by MCFLIRT by estimating mean displacement (30). Motion higher than 1.0 mm in rotation or translation was reason for exclusion. Images were spatially smoothed with a 6 mm full-width at half-maximum (FWHM) kernel to reduce noise without reducing valid activation. The boundary-based algorithm was used for registration (31). This requires both whole head and brain extracted input images. High resolution structural standard MNI space registration was performed using FLIRT (30, 32) and further refined with FNIRT nonlinear registration (12° of freedom and a 10 mm wrap resolution) (33, 34).

A first-level analysis was performed on each task run and included two regressors from the reward and control condition corresponding to the anticipation phase with the duration between 1 and 5 s. Time-series statistical analysis was carried out using FILM with local autocorrelation correction (35). Convolution was set to a double-gamma hemodynamic response function. Six motion parameters were included as regressors of no interest in addition to the derivatives of the original motion parameters and the squares of the derivatives, amounting to 18 regressors. Additional confound EVs were added to remove residual effects of motion that are still left in the data. For this we used the FSL tool called fsl_motion_outliers. This tool identifies outlier volumes or spikes in functional data and censors them based on certain criteria. For the current analysis, volumes were censored if framewise displacement was >0.9 (36). Runs were excluded from the analysis when more than 35 volumes were censored.

A second-level analysis was performed within subjects to combine the different runs using a fixed-effects model, by forcing the random effects variance to zero in FLAME (FMRIB's Local analysis of mixed effects) (37–39). These data were used for the group analysis to compare activity between MS and HC subjects ([MS anticipation – HC anticipation] and [MS control – HC control]). A whole-brain, voxel-wise independent samples *t*-test was performed using FLAME1 and 2 (38, 39). To correct for multiple comparisons, Z statistical images were thresholded using a cluster threshold of *z* > 2.3 and a corrected cluster significance threshold of p < 0.05 (40).

#### Structural MRI data

All T1-weighted images used for structural analyses for the MS group were first lesion-filled using FSL's lesion_filling protocol (41).

Segmentation of the hippocampus, caudate, putamen and nucleus accumbens was accomplished using FIRST (FSL v3.2.0), which is based on a Bayesian framework that allows for the relationships between the subcortical structures' sizes and shapes to be investigated. Linear registration using FLIRT (30–32) on T1-weighted images was performed in a two stage manner in which the images were first aligned on the MNI152 template with a 1 mm resolution using 12° of freedom, and then a subcortical mask was created in which excluded any unwanted regions outside of the subcortical structures being segmented. Once the mask was complete, all segmentation outputs were quality checked. Volumetric analyses were then performed on each structure bilaterally by using fslstats with the appropriate structure intensity. Volume was gained in mm^3^. SIENAX (40, 42), which is part of FSL (28), was then used to obtain a volumetric scaling factor per participant in order to normalize all brain tissue volume for differences in head size. SIENAX also performed tissue type segmentation, including estimates of white matter. Independent samples *t*-tests were then conducted between the region volumes of both groups.

Grey Matter (GM) volume using T1-weighted images was analyzed using FSL-VBM (43), an optimized VBM protocol (44) carried out with FSL tools (28). Structural images were registered to the MNI 152 standard space using non-linear registration (34) after brain-extraction and GM segmentation was completed. These images were then used to create a study-specific GM template which all native GM images were non-linearly registered to. All images were modulated to correct for local expansion/contraction and then smoothed with an isotropic Gaussian kernel with a sigma of 3 mm. Lastly, voxelwise general linear modeling was applied using permutation-based non-parametric testing. All tests were corrected for multiple comparisons across space.

#### Questionnaire data

Independent samples *t*-tests were performed on questionnaire data to see if there were differences between groups on the BIS/BAS scales. Pearson's correlations between questionnaire data and both subcortical volumes and averaged beta weights of the anticipation phase were then done to examine the relationships between motivational tendencies and both structure volume and activation between groups. For the MS group specifically, we controlled for disease duration by performing partial correlations.

Previous studies have shown that even with the use of tasks with non-contingent outcome presentation, individuals still often try to form strategies in order to feel more in control (45, 46). Thus, at the end of the experiment, we also asked participants about whether they used any strategies during task performance. Specifically, participants were asked the following question: “What strategies did you use while performing the task?” The questionnaire was coded as (1) if participants indicated using a strategy during the card task and [0] if participants indicated not using a strategy. The percentage of participants utilizing strategies was calculated for each group.

## Results

### Whole brain analysis

**Figure 2:**
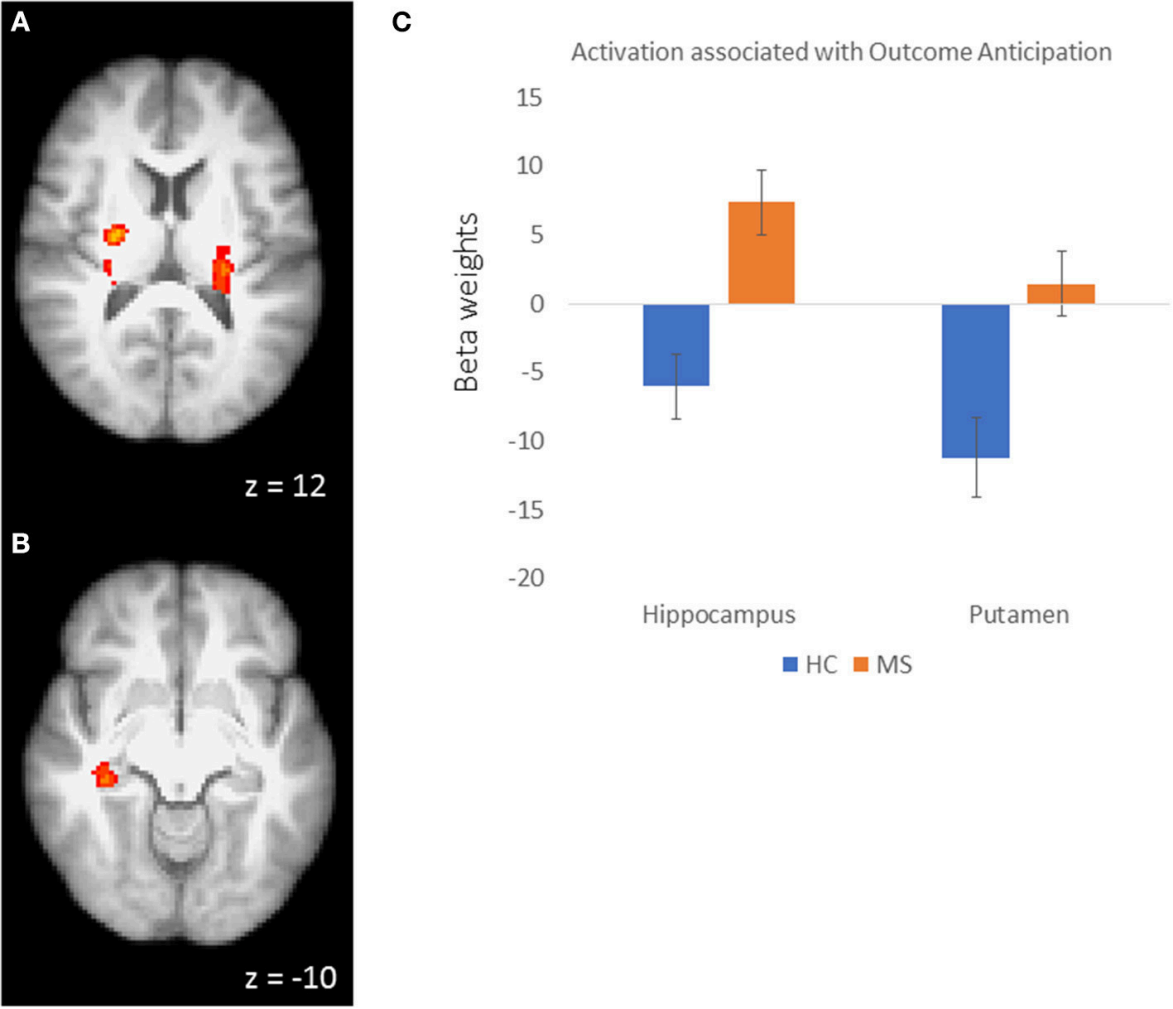
**(A)** Activation clusters in the putamen and thalamus during outcome anticipation in MS vs HC. **(B)** Activation cluster in the hippocampus during outcome anticipation in MS vs. HC. **(C)** For illustrative purposes, beta weights from the hippocampus and putamen clusters showing sensitivity during outcome anticipation in MS vs. HC.

In order to examine the neural mechanisms associated with outcome anticipation in individuals with MS, we performed an independent samples *t*-test, comparing activation during the anticipation periods of the reward and control conditions. In the reward condition, activations in the vmPFC was not different between MS and HC participants. However, we observed greater activation in the MS group than in the HC group in the right hippocampus, putamen and posterior cingulate cortex (PCC; Figure 2), among other regions (Table 2A; all *p*-values were corrected for cluster significance at *p* < 0.05 threshold). This finding replicates previous studies of clinical populations that showed increased hippocampal activation during outcome anticipation (43, 47). In the control condition, only areas of the occipital lobe and the right postcentral gyrus showed increased activation during outcome anticipation (Table 2B).

**Table 2:** **(A)** Brain regions showing increased activation in the MS group compared to the HC group during the anticipation period of the reward condition in MS vs. HC. **(B)** Brain regions showing increased activation in the MS group compared to the HC group during the anticipation period of the control condition.

**Region**	**Cluster size (mm^3^)**	**Hemisphere**	**Peak X**	**Peak Y**	**Peak Z**	**z-statistic**
**(A)**						
Cerebellum, anterior lobe	1,900	R	32	−58	−36	3.96
Cerebellum, posterior lobe	903	L	−18	−64	−48	3.54
Putamen/Hippocampus	737	R	26	−10	12	3.99
Posterior cingulate cortex (BA 31)	424	L	−8	−30	44	3.49
Thalamus	382	L	−22	−28	16	4.43
**(B)**						
Occipital cortex	1,710	R	46	−80	−10	3.55
Postcentral gyrus	587	L	−62	−28	46	3.94

### Volumetric analysis

A volumetric analysis of subcortical structures (deep GM) revealed significant bilateral differences of the hippocampus (right: *p* = 0.006; left: *p* = 0.017), caudate nucleus (right: *p* = 0.011; left: *p* = 0.004), and putamen (right: *p* = 0.017; left: *p* = 0.015) between groups, with the MS group having less volume than HCs on average. Additionally, the MS group showed significantly less volume in the left nucleus accumbens (*p* = 0.028) compared to HCs (see Table 3).

**Table 3:** Subcortical volumes per group.

	**Groups**		
	**HC**	**MS**	**Sig**
Hippocampus
Right	5626.93 (642.81)	4847.70 (652.80)	0.003
Left	5300.90 (526.30)	4726.09 (487.69)	0.004
Caudate nucleus
Right	4938.58 (542.04)	4312.20 (560.25)	0.005
Left	4814.23 (528.35)	4178.62 (456.59)	0.002
Putamen
Right	6892.99 (562.44)	6079.55 (819.06)	0.005
Left	6700.40 (644.96)	5896.45 (734.10)	0.009
Nucleus accumbens
Right	609.20 (167.89)	522.75 (181.26)	0.192
Left	743.21 (157.73)	611.57 (162.22)	0.034

*Data shown as Mean (Standard Deviation); Volumetric Data is shown in mm^3^*.

The VBM analysis showed no significant differences in cortical GM volume between groups. Significant white matter volume differences were observed (*p* = 0.006), with the MS group showing less white matter volume compared to HCs on average. All reported results were corrected for multiple comparisons.

### Brain-behavior relationships

We examined the relationship between brain parameters and participants' motivation levels. Specifically, we focused on areas in which MS participants showed greater activity than HCs during outcome anticipation: the putamen, hippocampus, and PCC. To start, independent samples *t*-tests revealed that MS participants and HCs showed no differences on either BIS (*p* = 0.71) or BAS (*p* = 0.29) questionnaires, indicating no differences in motivation levels. However, while controlling for disease duration, partial correlations revealed negative associations between BIS scores and activation of the putamen (*r*_*partial*_ = −0.633, *p* = 0.011), hippocampus (*r*_*partial*_ = −0.639, *p* = 0.010) and PCC (*r*_*partial*_ = −0.600, *p* = 0.018) in the MS group, but not with HCs. These results suggest that less aversive motivation is associated with greater activity in the putamen, hippocampus, and PCC during outcome anticipation in MS.

For the HC group, Pearson's correlations revealed a positive association between caudate volume and BAS scores (*r* = 0.700, *p* = 0.008), indicating that those who had more volume in their caudate nuclei also showed higher appetitive motivation.

### Strategy use during task performance

At the end of the experiment, we asked participants to indicate whether they used any strategies to predict the number on the face of the card. While outcome presentation during the task was random, some participants reported the use of strategies. Responses included statements such as “I tried to come up with a pattern,” “I tried to think of a pattern of numbers,” or “I was thinking about the pattern the examples showed.” Based on participants' self-reports about the use of strategies, we calculated the percentage of participants reporting the use of strategies during task performance. The results showed that 76% of participants in the MS group indicated that they used strategies during the card-guessing game in order to try to predict the outcome. Only 46% of participants in the HC group indicated that they used strategies during the task.

## Discussion

In the current study, we examined the neural mechanisms associated with outcome anticipation in individuals with MS. We observed that, compared to HCs, individuals with MS had reduced volumes of the striatal sub-regions and increased activation during outcome anticipation in the putamen. While we did not observe differences in the vmPFC activation, we observed greater activation in the right hippocampus and PCC during outcome anticipation in MS participants compared to their healthy counterparts. In addition, MS participants also exhibited reduced hippocampal volume compared to HCs.

### Putamen

In the current study, the outcomes of participants' guesses in the card-guessing task were predetermined to be 50% gains and 50% losses. That is, there were no patterns that participants could have learned to predict outcomes. However, 76% of the MS participants reported the employment of strategies while guessing during the reward condition to increase their gains, while only 46% of the healthy participants reported doing so. These results might suggest that MS participants are less aware of the lack of regularity between their guesses and the outcomes compared to healthy participants. In the current study, we found increased activation in the putamen during outcome anticipation in individuals with MS compared to HCs. Previous research has demonstrated that the putamen is involved in learning and outcome anticipation (48, 49). Thus, one potential explanation for the observed increased activation of the putamen in the MS, compared to HC, group might be that participants with MS were trying to use strategies and identify patterns in task structure, while HC participants were able to recognize a lack of regularity in the task structure. Failure to recognize a lack of task regularity might have resulted in stronger outcome anticipation, and thus greater activation in the putamen, as we observed in MS participants. From this perspective, the increased activation in the putamen might indicate impairment in learning about the reward structure, which manifested as redundant effort during outcome anticipation.

Another potential explanation for increased putamen activation is a lack of inhibition or impulsivity in the MS group. Previous studies have shown that individuals with MS show neural compensation during response inhibition by exhibiting higher activation in some brain regions including the putamen (50). Further, as previously discussed, individuals with MS have poor decision making processes and show less sensitivity to risky decisions (7–10). This could be a sign of impulsive or habitual behavior in MS. That is, increased putamen activation could also be associated with impulsivity, which is the tendency to select choices without forethought (51–53), or dominant habitual response, which is the proclivity to choose actions with no regard for their consequences (54). Interestingly, response inhibition, impulsive behavior and habitual responding share overlapping neural circuits that might be difficult to disentangle (55). Therefore, putamen activation in our MS sample could be a manifestation of these behaviors or due to patients' preparation for response (by using strategies to perform the cognitive task). More research is needed in order to tease apart these potential explanations of putamen involvement during decision-making and outcome anticipation in MS.

### Hippocampus and PCC

Structural and functional alterations of the hippocampus are well-documented in MS (56–58). Related to these previous findings, we observed differences in hippocampal activation during outcome anticipation between MS and healthy individuals. We discuss two possible explanations that may underlie the increased hippocampal activity in our MS group. First, the hippocampus may have been more active during outcome anticipation in order to compensate for the impaired activity in the striatum seen in MS. The hippocampus is a plausible candidate to cover for the striatum, as suggested by animal studies showing that the hippocampus and the striatum receive the same input information from dopaminergic midbrain areas (59). At the same time, it has been suggested that the hippocampus is involved in feedback learning and works in parallel with the frontal and striatal regions (60). Recent neuroimaging studies showed that hippocampal activation during outcome anticipation facilitates hippocampal coupling with the vmPFC (61). Furthermore, one study demonstrated that enhanced hippocampal activity compensates for the dysfunction of the caudate nucleus in patients with Huntington's disease (62).

Further, the hippocampus has been shown to play an important role in learning of regularities (63). As in the case of the putamen discussed above, the increased hippocampal activation might suggest that MS participants were actively engaging in determining regularities in task structure. As previously mentioned, participants with MS indicated trying to figure out a pattern to predict outcomes and maximize gains while performing the reward condition. Failing to learn the lack of regularity in the reward structure of the current task, MS participants might have tried harder to determine whether there were regularities, as individuals often do during gambling [e.g., gambler's fallacy and illusion of control 46, 64).

Third, together with the hippocampus, we also observed greater activation of the PCC in our MS group. The hippocampus and the PCC are part of the default mode network (DMN). This network has been shown to be deactivated during task performance, while activated during self-referential processing (65, 66). Reward processing during the card task can fall under the umbrella of self-referential processing as subjects have a goal to win monetary rewards for themselves. Thus, the current results might suggest that individuals with MS do not deactivate the DMN regions as do HCs during task performance. Such interpretation will be consistent with previous investigations of DMN activation in MS (67).

### Motivational tendencies

With the caudate nucleus being involved in anticipation and outcome processing (68), it comes as no surprise that we found a positive association between caudate volume and appetitive motivation in HCs. As for the MS group, a negative relationship was found between aversive motivation and the activation of the putamen, hippocampus, and PCC. That is, the more avoidant tendencies an individual has, the lower the activation of brain regions involved in outcome processing and learning of regularities, which goes hand in hand with the MS group being more strategic than the HCs. Furthermore, these observed relationships can be further interpreted as being due to impulsivity. Specifically, for the MS group, higher activation in the putamen might indicate a higher reflexive response, which means participants with MS may have responded to cards impulsively without motivation for the outcome in mind. With the HC group, however, more considerate responses should be made with motivation included in the thought process before selecting an action. While we did not observe functional differences in the caudate between groups, volumetric differences support this potential interpretation between appetitive motivation and caudate volume in the HC group. Future studies should utilize a paradigm which looks more closely at motivational aspects in order to more directly assess group differences in functional activation and individual differences in motivation.

## Limitations and future directions

Because our study is one of the first reports of structural and functional substrates related to outcome anticipation in MS, future studies should attempt to replicate our findings. This is particularly important since limited sample size and a homogenous gender profile of the sample (females only) are obvious limitations of the current study. While MS is more common in females [20–30% males 69), future studies should attempt to recruit a more representative sample.

Further, data collection for the current study did not entail any neuropsychological evaluations or assessment of the disability status (e.g., EDSS scores). Thus, future investigations of processes related to outcome anticipation should include the examination of the cognitive and disability profiles of MS patients to better characterize the sample. In addition, while we did not observe significant differences between groups in age, the HCs were somewhat older. Thus, future studies should better match the groups on age. Despite these limitations, the current study paves the way for further investigation of motivational tendencies and outcome anticipation and processing in individuals with MS as well as functional and structural correlates of these processes.

## Conclusion

The current study is the first to investigate functional architecture associated with outcome anticipation in individuals with MS. We observed (1) increased activation of several brain regions during outcome anticipation in MS compared to HCs, and (2) participants with MS not being able to identify the lack of regularity during the task and attempting to use strategies. While the present results require replication, they also add to the growing body of evidence suggesting structural and functional alterations of brain regions associated with anticipation and outcome processing in individuals with MS.

## Author contributions

ED conceived of the presented idea. AS, P-PL, and ED performed computations. AS, P-PL, JYN, EN, and ED contributed to final manuscript.

### Conflict of interest statement

The authors declare that the research was conducted in the absence of any commercial or financial relationships that could be construed as a potential conflict of interest. The reviewer PV and handling Editor declared their shared affiliation.
